# 1,25(OH)_2_D_3_ and VDR Signaling Pathways Regulate the Inhibition of Dectin-1 Caused by Cyclosporine A in Response to *Aspergillus Fumigatus* in Human Corneal Epithelial Cells

**DOI:** 10.1371/journal.pone.0164717

**Published:** 2016-10-18

**Authors:** Yiping Xia, Guiqiu Zhao, Jing Lin, Cui Li, Lin Cong, Nan Jiang, Qiang Xu, Qian Wang

**Affiliations:** 1 Department of Ophthalmology, The Affiliated Hospital of Qingdao University, Qingdao, Shandong, China; 2 Shandong Eye Institute, Qingdao, Shandong, China; University of Alabama at Birmingham, UNITED STATES

## Abstract

**Background:**

The objective of this study is to observe whether cyclosporine A (CsA) inhibits the expression of dectin-1 in human corneal epithelial cells infected with *Aspergillus fumigatus* (*A*. *fumigatus*) and to investigate the molecular mechanisms of the inhibition.

**Methods:**

Immortalized human corneal epithelial cells (HCECs) were pretreated with 1,25(OH)_2_D_3_ and VDR inhibitor for 1 h, and then they were pretreated with CsA for 12h. After these pretreatments, the HCECs were stimulated with *A*. *fumigatus* and curdlan respectively, and the expression of dectin-1 and proinflammatory cytokines (IL-1β and TNF-α) were detected by RT-PCR, western blot and ELISA.

**Results:**

Dectin-1 mRNA and dectin-1 protein expression increased when HCECs were stimulated with *A*. *fumigatus* or curdlan, and CsA inhibited the dectin-1 expression both in mRNA and protein levels specifically. Dectin-1 and proinflammatory cytokine expression levels were higher when HCECs were pretreated with VDR inhibitor and CsA compared to pretreatment with CsA alone, while dectin-1 and proinflammatory cytokine levels were lower when HCECs were pretreated with 1,25(OH)_2_D_3_ and CsA compared to pretreatment with CsA alone.

**Conclusions:**

These data provide evidence that CsA can inhibit the expression of dectin-1 and proinflammatory cytokines through dectin-1 when HCECs are stimulated by *A*. *fumigatus* or curdlan. The active form of vitamin D, 1,25(OH)_2_D_3,_ and VDR signaling pathway regulate the inhibition of CsA. The inhibition is enhanced by 1,25(OH)_2_D_3_, and the VDR inhibitor suppresses the inhibition.

## Introduction

Fungal keratitis is a rare but severe infectious inflammation of the cornea that is still one of the main causes of corneal blindness and visual disability[1].It occurs more frequently in developing countries with significant agriculture-dependent populations[2], such as India, Ghana, Nepal and China. In contrast, fungal keratitis is relatively uncommon in developed countries [3]. However, the treatment of fungal keratitis remains a great challenge for ophthalmologists today. *Aspergillus fumigatus* is known as one of the most common pathogens of fungal keratitis[4]. Pattern-recognition receptors (PRRs) that are expressed by innate immune cells recognize *Aspergillus fumigatus*.

Dectin-1 is one such innate immune PRR, having a transmembrane protein that contains a single C-type lectin-like domain (CTLD) in the extracellular region and an immunoreceptortyrosine-based activation (ITAM)-like motif within its intracellular tail[5].Through the recognition of β-glucans, dectin-1 can bind to several fungal species[6] and induce a vast array of cellular effects, including actin-mediated phagocytosis, activation of therespiratory burst through production of reactive oxygen species, endocytosis, dendritic cell maturation and production of cytokines such as TNF-α,IL-1β,CXCL2 and GM-CSF[7,8].

Cyclosporine A(CsA) is a calcineurin inhibitor that is widely used in ophthalmologic clinical treatment, such as for ocular allergy, infectious diseases and corneal transplantation[9–11]. During inflammation, CsA’s main function is to inhibit the phosphatase activity of calcineurin, which regulates nuclear translocation of the nuclear factor of activated T-cells (NFATs) transcription factor[12]. Impaired activation of NFATs then prevents the transcription of cytokine genes, including IL-1β and TNF-α[13].Recent studies have demonstrated that CsA can significantly down-regulate the expression of toll-like receptor 2(TLR2) and toll-like receptor 4(TLR4) in patients after liver transplantation [14].

Vitamin D is a fat-soluble vitamin[15] that is a secosterol produced endogenously in the skin from sun exposure or obtained from foods that naturally contain vitamin D, such as cod liver oil and fatty fish [16,17]. The active form of vitamin D, 1,25(OH)_2_D_3_, performs many of its biologic functions by regulating gene transcription through a nuclear high-affinity vitamin D receptor (VDR). Until recently, it was widely believed that vitamin D was solely activated through sequential hydroxylations by CYP27A1 or CYP2R1 at C25, and by CYP27B1 at C1 [18,19]: [D3→25(OH)D3→1,25(OH) 2D3]. This dogma has recently been refuted by the discovery that CYP11A1 hydroxylates D3, producing 20-hydroxyvitamin D3 [20(OH)D3]. The 20-hydroxyvitamin D3 can undergo further sequential hydroxylations of the side chain without its cleavage [20–23]: [D3→20(OH)D3→(OH) nD3]. The major metabolites of these pathways, such as 20-hydroxyvitamin D3 [20(OH)D3], 20-hydroxyvitamin D2 [20(OH)D2)], and 20,23-dihydroxyvitamin D3 [20,23(OH) 2D3], are biologically active [24] while being noncalcemic at pharmacological concentrations [25–28]. These novel hydroxy derivatives of vitamins D3 [29] and D2 [30] are also produced ex vivo in organs/cells expressing CYP11A1, including skin cells, where they would act as endogenous regulators [24].

This active metabolite of vitamin D binds to the nuclear VDR [31,32].Once bound, a variety of transcription factors attach to this complex, resulting in either up-or down-regulation of the gene’s activity[16,17,33]. Zbaobong Yin discovered that the mRNA of VDRs was expressed in human corneas [34]. Furthermore, CsA can inhibit the function of VDR[35], and the previous study demonstrated that the activation of VDR can decrease the expression of toll-like receptors [14]. However, the effect of CsA on dectin-1 is still unclear in human corneal epithelial cells, and the relationship between VDR and CsA has not been reported yet.

The influence of dectin-1 caused by CsA during *A*. *fumigatus* infection and the mechanism of this influence are still unknown. In this study, we investigated whether CsA can inhibit the expression of dectin-1 in human corneal epithelial cells when challenged with *A*. *fumigatus* ordectin-1 agonist curdlan.In addition, we provided evidence that the influence of dectin-1 caused by CsA is partially dependent on the function of VDR, offering a better understanding of CsA in the treatment of fungal keratitis.

## Materials and Methods

### Reagents

The *A*. *fumigatus* strain (NO3.0772) was bought from China General Microbiological Culture Collection Center. The Sabouraud culture was purchased from American Sigma Company. Trizol reagent, PrimeScript^®^ RT Reagent Kit with gDNA Eraser (Perfect Real Time), Primers and SYBR were purchased from TaKaRa. CsA(C8780) was purchased from Beijing Solarbio Science & Technology Co., Ltd. The rabbit anti-human VDR polyclonal antibody for western blot was purchased from Beijing Biosynthesis Biotechnology Co., Ltd. Polyclonal antibodies of dectin-1 (9051, dectin-1 antibody^®^) for western blot were purchased from Cell Signaling Technology. ZK195222 is a synthetic VDR antagonist, purchased from Bayer Pharma AG, Berlin German. ELISA DuoSet kits for human TNF-αand IL-1βwere purchased from BioLegend.

### Preparation of *Aspergillus fumigatus* Antigens

The *A*. *fumigatus* standard strain was grown on Sabouraud dextrose agar at 37°C and centrifuged at 200 rpm for 4 days. The mycelia collected after grinding was washed 3 times by sterile phosphate buffered saline (PBS) and disinfected by 75%ethanol at 4°C overnight. Then the inactivated *A*. *fumigatus* mycelia were washed 3 times and added in PBS. The myceliasuspensions were quantified by using a haemocytometer and stored at -20°C[36,37]. The final concentration of *A*. *fumigatus* mycelia was 1×10^8^/ml.

### Human Corneal Epithelial Cells Culture

Immortalized HCECs were cultured in high glucose medium, 37°C, 5% CO_2_. At nearly 90% confluence, the cells were cultured in serum-free Dulbecco's Modified Eagle medium (DMEM). Cells were used for real-time qPCR and western blot.

### Stimulation of *Aspergillus fumigatus* Antigens

Immortalized HCECs were cultured with CsA; the final solution of CsA was 1μM, 10μM, 100μM ina final ethanol concentration of 0.1%.Pretreatment of CsA was 12h before *A*. *fumigatus* stimulation; final *A*. *fumigatus* stimulation liquid was 5×10^6^/ml. The expression of dectin-1 and proinflammatory cytokines mRNA in HCEC was detected by real-time polymerase chain reaction (qPCR) 8h after *A*. *fumigatus* stimulation.

### Stimulation of VDR Inhibitor or 1,25(OH)_2_D_3_

HCECs were pretreated with VDR inhibitor (final concentration: 0.2μM/) or 1,25(OH)_2_D_3_ (final concentration: 100nM) respectively for 1 h before being stimulated with CsA and *A*. *fumigatus*. Expression of dectin-1 and proinflammatory cytokines were detected.

### Real-time qPCR

Total RNA was isolated from cells by using total RNA extraction reagent (RNAiso PLUS) solution and quantified by spectrophotometry. β-actin is the housekeeping gene. Quantitative real-time PCR was performed using the Eppendoft Mastercycler and SYBR green. Primers shown in Table 1 were used.

**Table 1:** Primers.

Gene Name	Primer Sequence(5’-3’)
***β-actin***	F-GCT GAT GGC CCT AAA CAG ATG AA
	R-TCC ATG GCC ACA ACA ACT GAC
***Dectin-1***	F-CGACTCTCAAAGCAATACCAGGA
	R-GTACCCAGGACCACAGCTATCAC
***TNF-α***	F-CAG TGG GCT GAT TAG AGA GAG GT
	R-TGC TTG TTC CTC AGC CTC TT
***IL-1β***	F-GCT GAT GGC CCT AAA CAG ATG AA
	R-TCC ATG GCC ACA ACA ACT GAC

Applied recommend steps in PrimeScript RT Reagent Kit with gDNA Eraser were used as reverse transcription steps: 95°C for 10 min, followed by 40 cycles of 95°C for 20 sec, and 65°C for 45 sec. Flourescence values were recorded at the end of each cycle reaction. The 40 cycles formed an amplification curve and reported the number of cycles that reached the threshold fluorescence value (Ct value).Values are expressed as ratios of fold change of dectin-1 and proinflammatory cytokines.

### Western Blot

The cell lysate (50μg per lane, measured by the BCA protein assay kit) was mixed with 6X SDS-PAGE Sample Loading Buffer and then boiled for 10min before loading it into the gel. The proteins were separated bymolecular weight marker on SDS polyacrylamide 10% gel and transferred to polyvinylidene difluoride (PVDF) membranes for 1 h in 100 V in the blotting system for western blot analysis. The membranes were blocked with 5% bovine serum albumin (BSA) blocking buffer for 1 h at 37°C with gentle shaking. Dectin-1 primary antibody was diluted 1,000 times by using primary antibody diluents, and the secondary antibody was diluted 2,500 times in phosphate buffered saline (PBS). The membranes were first incubated with primary antibody of dectin-1 overnight at 4°C, then incubated with the secondary antibody, for 1 h at 37°C. The signal bands were detected with BeyoECL Plus chemiluminescence reagent using a UVP EC3 Imaging System.

### Enzyme-Linked Immunosorbent Assay

Double antibody sandwich ELISA for human TNF-α and IL-1βwas performed according to the manufacturer’s protocol from BioLegend, to determine the protein concentration of different treatments. Absorbance was read at 450 nm with a reference wavelength of 570 nm by a VERSA max microplate reader(Molecular Devices, Sunnyvale, CA).The plate was coated with 100 μl diluted Capture Antibody incubated overnight at 4°C. We blocked non-specific binding and reduced background with Assay Diluent A for 1 h with shaking. After adding 50ul Assay Buffer D with the diluted standard and samples, the plate was sealed and incubated at room temperature for 2 h with shaking. Then the diluted detection antibody was added to each well for 1 h before incubation with diluted Avidin-HRP solution for 30 min. As a final step, the plate was treated with Substrate Solution F and incubated in the dark for 20 min. The reaction was terminated using stop solution 2N H_2_SO_4_. Absorbance was read at 450 nm with a reference wavelength of 570 nm by a VERSA max microplate reader.

### Statistical Analysis

All experiments were repeated at least three times. Each sample was minimally run in triplicate, and all data were presented as mean±SD from independent experiments. The data were analyzed using SPSS19.0.A one-way ANOVA test was used to comparethree or more groups, and Fisher’s least significant difference (LSD) was used to identify significant differences between the two groups. Statistical significance was set at P<0.05.

## Results

### The increase of Dectin-1 mRNA and protein was inhibited by CsA when HCECs were pretreated with CsA before *A*. *fumigatus* and curdlan stimulation

**Fig 1:**
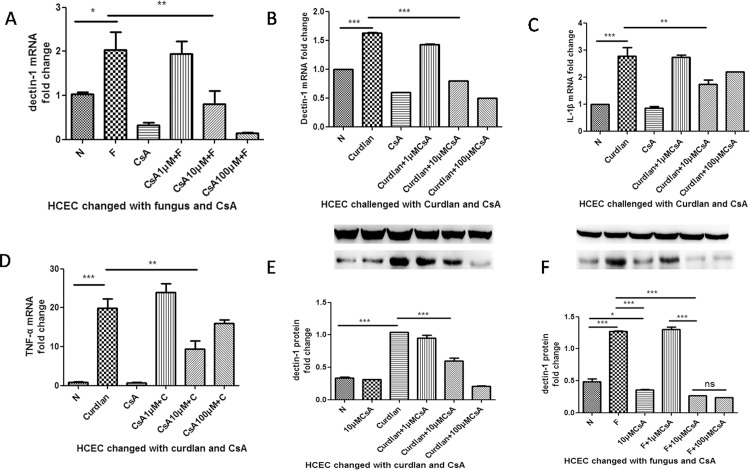
**(A-F) The mRNA and protein fold change of dectin-1 and proinflammatory cytokines when HCECs were challenged with CsA and *A*. *fumigatus*.** The expression of dectin-1 and proinflammatory cytocines decreased when stimulated by CsA. N in each graph represents the control group, and F represents the fungal-stimulation group. The mRNA fold change (A) and protein fold change (E) of dectin-1 were significantly inhibited when pretreated with CsA before stimulation of *A*. *fumigatus*. B shows dectin-1 expression when stimulated with curdlan. The mRNA fold change (B) and protein fold change (F) of dectin-1 is also inhibited significantly. C and D were the mRNA fold change of proinflammatory cytokines when challenged with curdlan, TNF-α (C) and IL-1β (D) are both inhibited significantly.

During our preliminary study, CsA in the concentration 10μM was optimal. Antifungal immunity induced by dectin-1 occurs after recognition of the pathogenic *A*. *fumigatus* or its specific ligand, β-glucan curdlan, resulting in the up-regulation of dectin-1 expression. We pretreated the HCECs with CsA of 1μM, 10μM and 100μMfor 12 hand then stimulated them with the *A*. *fumigatus* or curdlan for 8 h. Stimulation of HCECs by *A*. *fumigatus* significantly increased the mRNA expression of dectin-1(Fig 1A*P<0.05), and dectin-1 expression canalso be induced by curdlan (Fig 1B***P<0.001). Then HCECs were challenged with CsA for 12 h before *A*. *fumigatus* or curdlan stimulation. Dectin-1 mRNA expression in response to *A*. *fumigatus* (Fig 1A**P<0.01) or curdlan (Fig 1B***P<0.001) was obviously abrogated by CsA. The protein expression level was also tested. Our results showed that CsA inhibits dectin-1 protein expression when stimulated by *A*. *fumigatus* (Fig 1F ***P<0.001) or curdlan (Fig 1E***P<0.001).

CsA inhibited proinflammatory cytokine expression through its classical NFATs pathway in response to *A*. *fumigatus*[38]. Thus, we further investigated whether the expression of proinflammatory cytokines (TNF-αand IL-1β) mRNA can be inhibited through a dectin-1 signaling pathway when challenged by dectin-1 agonist curdlan. We found that TNF-α (Fig 1C**P<0.01) and IL-1β(Fig 1D**P<0.01) production were significantly inhibited by CsA.

### The Effect of CsA on Dectin-1 Can Be Suppressed by the Stimulation of a VDR Inhibitor

**Fig 2:**
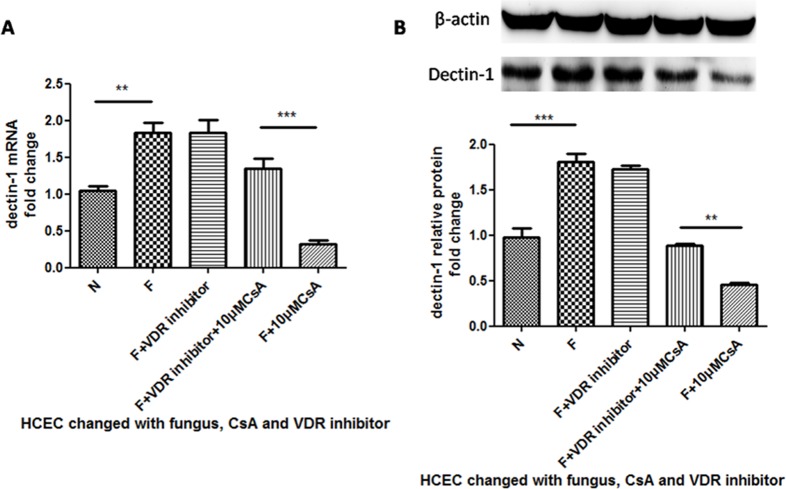
**(A-B) The change of dectin-1mRNA and protein expression when HCECs were pretreated with VDR inhibitor.** We pretreated the HCECs with VDR inhibitor for 1 h before CsA and 13 h before *A*. *fumigatus* stimulation. A was the mRNA fold change of dectin-1. B was the relative protein change of dectin-1.

We next set out to elucidate how CsA stimulation inhibited the expression of dectin-1 and proinflammatory cytokine expression. Because VDR was associated with PRR expression and CsA influenced the function of VDR[35], we next explored the relationships among CsA, VDR and dectin-1 expression. HCECs were pretreated with a VDR inhibitor(concentration of 0.2μM) for 12 h before the stimulation of *A*. *fumigatus*. As a control, the VDR inhibitor stimulation group had no differences of dectin-1 mRNA expression after treatment of *A*. *fumigatus* (Fig 2A). The group pretreated with VDR inhibitor before CsA stimulation weakened the inhibition of CsA significantly compared with the group stimulated with CsA alone(Fig 2A***P<0.001), which suggested VDR blocking suppressed the inhibition effect of CsA in response to *A*. *fumigatus*. The protein expression change showed that pretreatment of VDR inhibitor significantly weakened the inhibition of CsA, which is consistent with the mRNA fold change (Fig 2B **P<0.01).

### The Effect of CsA on Proinflammatory Cytokines Was Suppressed when HCECs Were Stimulated by VDR Inhibitor

**Fig 3:**
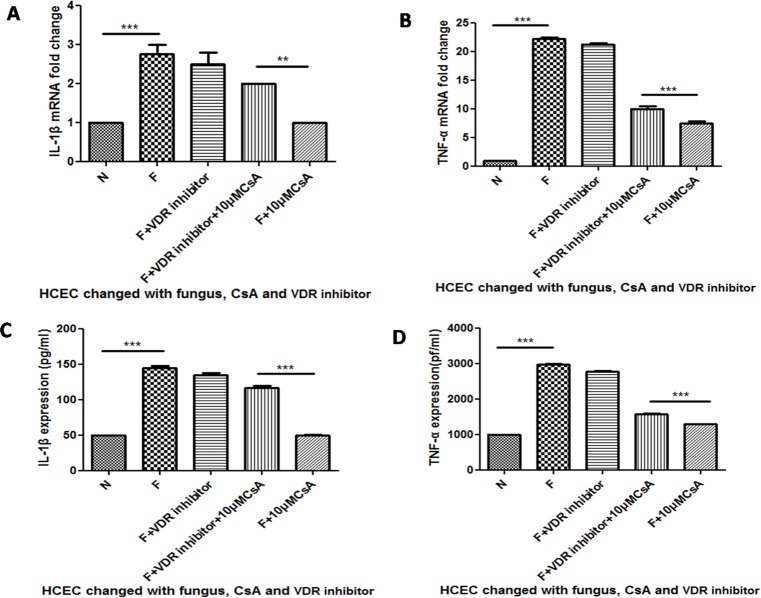
The change of IL-1β and TNF-α mRNA and protein expression when HCECs were pretreated with VDR inhibitor. We pretreated the HCECs with VDR inhibitor for 1 h before CsA and 13 h before *A*. *fumigatus* stimulation. A was the RNA fold change of IL-1β. C was the protein change of IL-1β. The IL-1β inhibition of the group pretreated with VDR inhibitor was significantly weakened compared to the group without VDR inhibitor stimulation. The same change could be seen in TNF-α (B and D).

Proinflammatory cytokines TNF-α and IL-1βwere produced through the dectin-1 signaling pathway, and we have shown that CsA can inhibit dectin-1 expression, so we hypothesized that CsA can influence TNF-α and IL-1β expression through the dectin-1 pathway. To further investigate the role of CsA in the dectin-1 pathway during *A*. *fumigatus*infection, TNF-α and IL-1β were detected in mRNA and protein level (Fig 3). A higher level than normal of IL-1β mRNA was detected with *A*. *fumigatus* stimulation (Fig 3A***P<0.001), and CsA inhibited the up-regulation (***P<0.001). When HCECs were pretreated with VDR inhibitor, the inhibition of CsA was significantly weakened (**P<0.005). The protein levels change was greater than the mRNA levels change when pretreated with VDR antibody (***P<0.001).The results of TNF-α mRNA and protein levels were also significantly changed (Fig 3B and 3D***P<0.001).

### The Inhibition of Dectin-1 Can Be Enhanced by 1,25(OH)_2_D_3_ Stimulation

**Fig 4:**
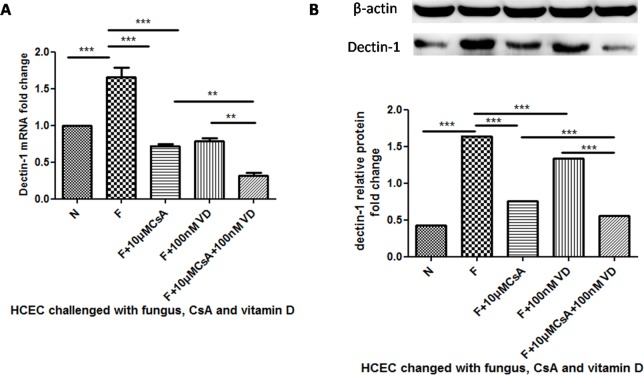
The change of dectin-1 mRNA and protein expression when HCECs were challenged with 1,25(OH)_2_D_3_ before *A*. *fumigatus* and CsA stimulation. We pretreated the HCECs with 1,25(OH)_2_D_3_ for 1h before CsA and 13 h before *A*. *fumigatus* stimulation. A depicts the mRNA fold change of dectin-1. Dectin-1 inhibition of the group pretreated with 1,25(OH)_2_D_3_ was significantly enhanced compared to the group without 1,25(OH)_2_D_3_stimulation. The same change could be seen in the protein expression of dectin-1 (B).

We next investigated whether 1,25(OH)_2_D_3_, which activates VDR, had the opposite function of the VDR antibody. HCECs were pretreated with 1,25(OH)_2_D_3_ in the concentration of 100nM for 1 h and CsA in the concentration of 10μM for 12 h respectively before the stimulation of *A*. *fumigatus*. The mRNA expression of dectin-1 was inhibited when HCECs were challenged with both CsA and 1,25(OH)_2_D_3_(Fig 4A***P<0.001). When the HCECs were pretreated with1,25(OH)_2_D_3_ for 1 h before the stimulation of CsA and *A*. *fumigatus*, CsA inhibition was enhanced significantly(Fig 4A**P<0.01). Moreover, dectin-1 protein expression was tested to prove the mRNA fold change, demonstrating that 1,25(OH)_2_D_3_ did enhance CsA inhibition (Fig 4B***P<0.001).

### The Inhibition of Proinflammatory Cytokines Was Enhanced when HCECs Were Stimulated by 1,25(OH)_2_D_3_

**Fig 5:**
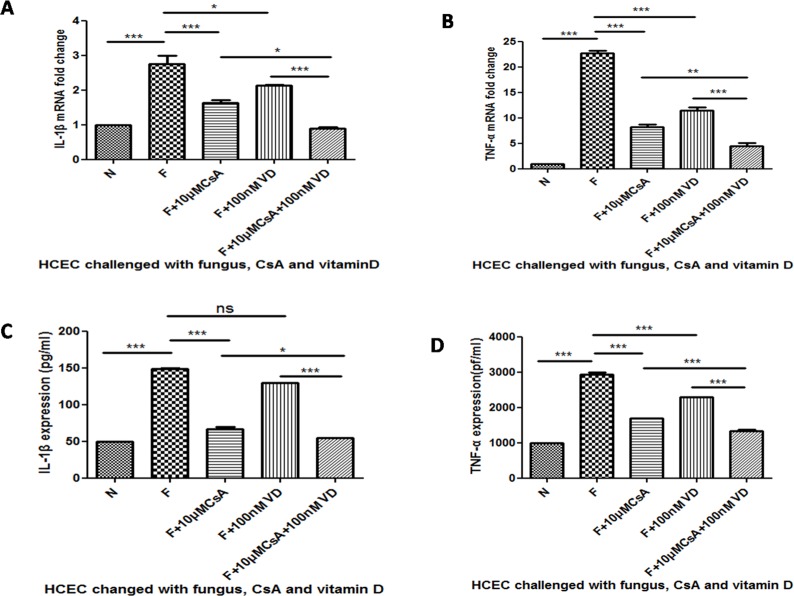
The change of IL-1β and TNF-α mRNA and protein expression when HCECs were pretreated with vitamin D. We pretreated the HCECs with1,25(OH)_2_D_3_ for 1 h before CsA and 13 h before *A*. *fumigatus* stimulation. A was the mRNA fold change of IL-1β. C was the protein change of IL-1β. The IL-1β inhibition of the group pretreated with 1,25(OH)_2_D_3_ was significantly enhanced compared to the group without 1,25(OH)_2_D_3_ stimulation. The same could be seen in the change of TNF-α (B and D).

To further investigate the regulation of vitamin D in *A*. *fumigatus* infection, we pretreated the HCEC with 1,25(OH)_2_D_3_ for 1 h before CsA and 13 hbefore *A*. *fumigatus* stimulation and then tested the mRNA and protein expression of TNF-α and IL-1β. The results demonstrated that *A*. *fumigatus* up-regulated the mRNA expression of IL-1β (Fig 5A***P<0.001). CsA inhibited up-regulation (***P<0.001). The pretreatment of 1,25(OH)_2_D_3_ enhanced the inhibition (*P<0.05). The protein levels change and mRNA level change had equal significance (Fig 5C*P<0.05). The 1,25(OH)_2_D_3_ treatment also enhanced TNF-α mRNA (Fig 5B**P<0.005) and protein level changes(Fig 5D ***P<0.001).

## Discussion

CsA is a powerful immunosuppressive and immunomodulatory drug widely used in ophthalmic clinical practice. Its efficacy to treat vernal keratoconjunctivitis was detected in 1986. In addition, it has been used as a prescription drug since 2003 in the United States to treat dry eye syndrome [39] and as rejection prophylaxis and treatment in corneal grafts [40]. CsA has an anti-inflammatory function through inhibiting the phosphatase activity of calcineurin to decrease the production of proinflammatory cytokines[12]. Although related studies have reported that CsA can down-regulate the expression of TLR2 and TLR4 [14], the influence of CsA on pattern recognition receptor dectin-1 remains unclear.

In our study, dectin-1 expression and proinflammatory cytokine production were increased when HCECs were stimulated with *A*. *fumigatus* and dectin-1 agonist curdlan. CsA pretreatment inhibited this up-regulation (Fig 1). Our previous laboratory studies showed that *A*. *fumigatus* up-regulated the expression of TLR2, TLR4 and dectin-1 in the corneal epithelium [41]. In addition, curdlan, as the agonist of dectin-1, was also able to increase the expression of dectin-1receptors in monocytes[39]. Meanwhile, a therapeutic dose of CsA resulted in a marked down-regulation of TLR2, TLR4 and MyD88, and it decreased macrophage infiltration at 48 h after rat cardiac transplantation[38]. Also, CsA had an inhibitory effect on the TLR4 signaling pathway and decreased proinflammatory cytokines production when stimulated with a TLR4 agonist [14]. Although CsA is known to influence the expression and function of TLRs, the exact effect on dectin-1 expression has remained elusive. According to our data, dectin-1, as a PRR, was inhibited by CsA stimulationand cytokine production stimulated by dectin-1 agonist curdlan decreased significantly as well (Fig 1), which is consistent with previous studies. However, in the study of cyclosporine-induced renal injury, long-term CsA treatment was able to up-regulate TLR2 and TLR4 mRNA and protein expression on renal tubular cells, which was inconsistent with our results[42].This inconsistency might be due to the difference in treatment time. In our study, HCECs were treated with CsA for12 h, but in a previous study the experiment rats were cultured for at least two months. Long-term treatment might increase PRR expression.

In our study, CsA inhibited dectin-1 expression, but when HCECs were pretreated with VDR inhibitor, the inhibition was impaired significantly (Fig 3). In contrast to the VDR inhibitor impairment, inhibition by VDR activator 1,25(OH)_2_D_3_ enhanced stimulation. According to these data, we speculated that the influence of CsA on dectin-1 might be related to the function of VDR. VDR activator 1,25(OH)_2_D_3_ down-regulated TLR2 and TLR4 expression, compared to up-regulation of TLR10. When blocked the VDR with VDR inhibitor, the up-regulation of TLR10 was significantly blocked [40]. In addition, there is an impairment function of the VDR pathway by CsA in the study of a Wistar rat’s kidney[35]. These data suggest that VDR participates in the regulation of PRR expression and that CsA can influence VDR function, which is in line with our hypothesis and experiment data.

In addition, our results also show the fold change of proinflammatory cytokine production and secretion. Proinflammatory cytokine mRNA production increased significantly when stimulated with curdlan, and it was inhibited when stimulated with CsA. In order to test the relationship between CsA and VDR, HCECs were stimulated to examine the production and secretion of TNF-α and IL-1β after VDR inhibitor and 1,25(OH)_2_D_3_ stimulation. As a result, the same tendency was observed with dectin-1 expression. CsA can inhibit the production of pro-inflammatory cytokines such as TNF-α, IL-1β, IL-6 and IL-8 through its classical NFATs pathway[12]. Also, CsA had an inhibitory effect on the TLR4 signaling pathway and decreased pro-inflammatory cytokine production induced by the TLR4 signaling pathway when stimulated with a TLR4 agonist [14]. Our data demonstrated that CsA also has an inhibitory effect on proinflammatory cytokine production through dectin-1.

In summary, as an immunosuppressant, CsA inhibits pattern recognition receptor dectin-1 expression and inhibits its function, which controls pro-inflammatory cytokine production through dectin-1 pathway. The function of VDR and 1,25(OH)_2_D_3_ may be directly related to CsA inhibition. This has important implications for prophylaxis and treatment of fungal keratitis.

It is worthwhile to use animal models to study the effects of CsA on dectin-1 expression with long-term CsA pretreatment.

## Supporting Information

S1 File This file is the primary data. (XLSX)
